# Effects of salinity and drought on growth, ionic relations, compatible solutes and activation of antioxidant systems in oleander (*Nerium oleander* L.)

**DOI:** 10.1371/journal.pone.0185017

**Published:** 2017-09-18

**Authors:** Dinesh Kumar, Mohamad Al Hassan, Miguel A. Naranjo, Veena Agrawal, Monica Boscaiu, Oscar Vicente

**Affiliations:** 1 Instituto de Biología Molecular y Celular de Plantas (UPV-CSIC), Universitat Politècnica de València, Valencia, Spain; 2 Department of Botany, University of Delhi, Delhi, India; 3 Instituto Agroforestal Mediterráneo, Universitat Politècnica de València, Valencia, Spain; RIKEN Center for Sustainable Resource Science, JAPAN

## Abstract

*Nerium oleander* is an ornamental species of high aesthetic value, grown in arid and semi-arid regions because of its drought tolerance, which is also considered as relatively resistant to salt; yet the biochemical and molecular mechanisms underlying oleander’s stress tolerance remain largely unknown. To investigate these mechanisms, one-year-old oleander seedlings were exposed to 15 and 30 days of treatment with increasing salt concentrations, up to 800 mM NaCl, and to complete withholding of irrigation; growth parameters and biochemical markers characteristic of conserved stress-response pathways were then determined in stressed and control plants. Strong water deficit and salt stress both caused inhibition of growth, degradation of photosynthetic pigments, a slight (but statistically significant) increase in the leaf levels of specific osmolytes, and induction of oxidative stress—as indicated by the accumulation of malondialdehyde (MDA), a reliable oxidative stress marker—accompanied by increases in the levels of total phenolic compounds and antioxidant flavonoids and in the specific activities of ascorbate peroxidase (APX) and glutathione reductase (GR). High salinity, in addition, induced accumulation of Na^+^ and Cl^-^ in roots and leaves and the activation of superoxide dismutase (SOD) and catalase (CAT) activities. Apart from anatomical adaptations that protect oleander from leaf dehydration at moderate levels of stress, our results indicate that tolerance of this species to salinity and water deficit is based on the constitutive accumulation in leaves of high concentrations of soluble carbohydrates and, to a lesser extent, of glycine betaine, and in the activation of the aforementioned antioxidant systems. Moreover, regarding specifically salt stress, mechanisms efficiently blocking transport of toxic ions from the roots to the aerial parts of the plant appear to contribute to a large extent to tolerance in *Nerium oleander*.

## Introduction

Environmental abiotic stress factors, especially drought and soil salinity, are the major causes for reduction of agricultural yields worldwide [1,2]. The forecasted consequences of global climate change—higher mean temperatures, changes in seasonal weather patterns, increased frequency, intensity and duration of drought periods and ‘heat waves’, increasing scarcity of water for irrigation, etc.—will no doubt worsen this problem in the coming years, at least in arid and semiarid regions [3]. This situation has boosted research on the mechanisms plants activate to respond to water, salt and other abiotic stresses; knowledge gained from these studies will contribute to design and implement strategies for the genetic improvement of crop stress tolerance by both, classical breeding and genetic engineering approaches [4–7]. For obvious reasons, such studies have mostly focused on our major cultivated species—apart from model plants such as *Arabidopsis thaliana*–thus neglecting minor crops (and wild species) with a wide range of tolerance levels, which could provide relevant additional information on general stress tolerance mechanisms in plants. Although those species are grown at a much lower scale than major food, feed or fiber crops, many are still economically important because of their ornamental, medicinal, aromatic, cosmetic, culinary or other industrial uses.

*Nerium oleander* L. (fam. Apocynaceae) is an evergreen shrub native to the Mediterranean region and the Middle East, commonly used as an ornamental and medicinal plant. In the Iberian Peninsula, it is very frequent in dry and semi-arid habitats up to altitudes of 600 m, as well as along unstable water courses where short periods with abundant water flow alternate with long phases of more or less intense drought, in which the level of the water table falls sharply. Plants growing in this type of habitats not only suffer severe drought episodes, but also the effects of soil erosion caused by the torrential character of the precipitations and abrasion by the drag of sand and gravel. In addition, the concentration of chlorides and sulfates in the soil is usually higher than in the neighboring contiguous zones because of the ascendant washing of mineral salts [8].

*Nerium oleander* is frequently grown as an ornamental plant in arid regions, since it requires little water and not much maintenance, admits pruning operations, and is a continuous bloomer producing flowers throughout summer and early autumn. Oleander has a high aesthetic value due do to its variegated leaves and its large and colorful flowers. Over 400 different cultivars are grown in warm areas, in landscapes, gardens, parks, and along roadsides; oleander’s use as ornamental is limited only by its sensitivity to cold temperatures and frost. Cultivated genotypes vary in their morphological traits, yet the species shows little variability in the wild, as it is self-compatible and seldom cross-pollinated by insects [9]. Although its native distribution area is wide, only one subspecies from the eastern Mediterranean—*N*. *oleander* subsp. *kurdikum* Rech.—has been spilt from the typical subspecies, *N*. *oleander* subsp. *oleander*.

Oleander’s leaf structure is a classic example of xeromorphic anatomic adaptations, which are believed to be partly responsible for the well-known resistance to drought of this species. The leathery leaves are covered with a thick cuticle, and the stomata are located on the lower surface of the leaves and sunk in depressions covered by microscopic hairs so that water loss is reduced [10]. There are some publications describing changes in physiological parameters—growth, gas exchange, water relations, chlorophyll fluorescence, etc.—in water-stressed oleander plants. For example, it has been reported that plants submitted to water deficit for 10 days showed only minor variations in leaf gas exchange, while the leaf water content remained unchanged even 22 days after ceasing irrigation; plants survived one-month drought treatments, even though they lost their ornamental value, but could completely recover when water was supplied again [11]. Apart from these anatomical and physiological data, there is still very limited knowledge on the responses of this species to water stress and the biochemical and molecular mechanisms underlying its drought tolerance. Even less information is available on oleander’s responses to high soil salinity. In a general study including several landscape species, oleander was classified as ‘salt tolerant’, based on the symptoms produced by sprinkler irrigation with saline water [12]. It has even been considered as a halophyte by its inclusion in a study dealing with the possible use of halophytes for phytoremediation of heavy metal-contaminated soils [13]. There are also data indicating that plants fertilized with nitrate (as opposed to ammonium) as nitrogen source are more tolerant to salinity and show lower Na^+^ and Cl^-^ shoot contents, suggesting the occurrence of mechanisms blocking ion transport to the aerial part of the plants, which may contribute to tolerance [14]. Yet, to our knowledge, no systematic study on oleander’s responses to salinity and its putative biochemical and molecular mechanisms of salt tolerance has been published up to now.

This study was conducted to analyze different physiological and biochemical responses of one-year-old *N*. *oleander* seedlings to controlled salt and drought stress treatments, to try and establish which of those responses are more relevant for stress tolerance. All plants activate a number of common mechanisms and reactions when affected by water deficit, soil salinity and other abiotic stresses; these responses generally include, among others, inhibition of growth, degradation of photosynthetic pigments, regulation of ion transport, biosynthesis and accumulation of specific osmolytes for osmotic adjustment, or the activation of antioxidant systems—since the aforementioned stressful conditions cause secondary oxidative stress in plants [2,15–18]. In our experiments we included ‘shock treatments’ at very high salt concentrations and by completely withholding irrigation for up to 30 days to check how plants could respond to increased stress conditions in their natural environments—as expected under the foreseeable effects of climate change—and to assess the possibility to use low quality, saline water to grow cultivated oleander plants.

With this aim, we have analyzed how growth inhibition of oleander plants in response to water deficit and salt treatments correlates with the levels of ***i)*** photosynthetic pigments, ***ii)*** monovalent ions (Na^+^, K^+^ and Cl^-^), ***iii)*** different osmolytes commonly used by plants (proline, glycine betaine and soluble sugars), ***iv****)* oxidative stress [using malondialdehyde (MDA) as a lipid peroxidation biomarker], and *v****)*** several enzymatic and non-enzymatic antioxidants.

## Material and methods

### Plant material and stress treatments

One-year-old seedlings of *N*. *oleander*, grown from seeds sampled in the wild, were provided by the Center for Forest Research and Experimentation (CIEF), Valencia Community. The seedlings were transplanted to a nutritive substrate of peat (50%), perlite (25%) and vermiculite (25%), and kept moistened with half-strength Hoagland solution [19]. The newly transplanted seedlings were allowed to acclimatize for one week in the greenhouse before starting the stress treatments, watering them twice per week with the nutrient solution. Salt stress was applied by supplementing the Hoagland solution with NaCl to final concentrations of 200, 400 or 800 mM. Water stress treatments were performed by completely withholding irrigation of the pots. Ten individual plants were used for each of the five different treatments (control, water-stress and three NaCl concentrations); five plants, representing biological replicas for each treatment, were harvested after 15 days and the remaining five after 30 days of treatment.

### Plant growth parameters

The relative stress-induced inhibition of vegetative growth was estimated by determining a number of growth parameters in the control and the stressed plants, after 15 and 30 days of treatment: stem length (SL) elongation (referred to the stem length of each plant at time 0), leaf fresh weight (FW), dry weight (DW), and water content percentage (WC%). After measuring total leaf FW for each plant, a fraction of the sample was weighed, dried at 65°C for 72 hours and weighed again to determine DW. The water content percentage in leaves was calculated as: WC% = [(FW − DW)/FW] x 100. Fresh leaf material to be used for antioxidant enzyme assays was flash-frozen in liquid N_2_ and stored at -75°C; dry material was stored at room temperature in tightly closed tubes. Root samples of all plants were also collected, weighed, dried and stored as indicated for the leaf samples.

### Photosynthetic pigments

Photosynthetic pigments in the leaves of harvested plants were quantified after extraction with acetone [20]. Chlorophyll a (Chl a), chlorophyll (Chl b), and total carotenoids (Caro) were extracted from 0.1 g of fresh leaf material by grinding in the presence of 30 mL ice-cold 80% acetone; the sample was gently mixed overnight in an orbital shaker, centrifuged, and the absorbance of the supernatant was determined at 663, 646, and 470 nm. Pigment concentrations were calculated using published equations [20], and were expressed as ‘mg g^-1^ DW’.

### Monovalent ions contents

Contents of sodium, chloride and potassium in shoots and roots of the harvested plants were determined in aqueous extracts, prepared by incubating the samples (0.1 g of dried, ground leaf or root material in 15 mL of water) for 1 h at 95°C, and followed by filtration through a 0.45 μm nylon filter [21]. Na^+^ and K^+^ were quantified with a PFP7 flame photometer (Jenway Inc., Burlington, USA), and Cl^-^ was measured using a Merck Spectroquant Nova 60 spectrophotometer and its associated test kit (Merck, Darmstadt, Germany).

### Osmolyte determination

Proline (Pro) content was determined using dry leaf material, according to the previously published ninhydrin-acetic acid method [22]. Extracts were prepared in 3% aqueous sulfosalicylic acid, mixed with acid ninhydrin solution, incubated for 1 h at 95°C, cooled on ice and then extracted with toluene. After centrifugation, absorbance of the organic phase was read at 520 nm using toluene as a blank. Pro concentration was expressed as μmol g^-1^ DW. Glycine betaine (GB) concentration in leaves was determined according to a previously published procedure [23]. Dry leaf material (0.1 g) was ground with 2 ml of ice-cold Milli-Q water, mixed with potassium iodide, kept on ice for 90 min and then extracted with 1, 2-dichlorethane (pre-cooled at -20°C); finally, the absorbance of the solution was measured at 365 nm. GB concentration was expressed as μmol·g^−1^ DW. Total soluble sugars (TSS) were quantified in methanol extracts [0.1 g dry leaf material ground in the presence of 3 mL 80% (v/v) methanol]; the sample was mixed on a rocker shaker for 24 h and centrifuged; the supernatant was collected, concentrated sulfuric acid and 5% phenol was added and the absorbance of the resulting solution was finally determined at 490 nm [24]. TSS content was expressed as ‘mg equivalent of glucose’ per g DW.

### MDA and non-enzymatic antioxidants

Malondialdehyde (MDA), total phenolic compounds (TPC), and total flavonoids (TF) were quantified in the same methanol extracts used for TSS determination. Leaf MDA contents were determined as previously described [25], with minor modifications. Methanol extracts were mixed with 20% trichloroacetic acid (TCA) and 0.5% thiobarbituric acid (TBA), and incubated at 95°C for 15 min. The sample was centrifuged and the absorbance of the supernatant was measured at 600 and 540 nm; MDA concentration was calculated using the equations included in [25]. TPC were quantified according to [26], by measuring the absorbance of the methanol extracts at 765 nm after reaction with the Folin-Ciocalteu reagent; TPC contents were expressed as equivalents of gallic acid (mg eq GA g^-1^ DW), used as a standard. TF were measured following the procedure described in [27], based on the nitration of aromatic rings bearing a catechol group and their reaction with AlCl_3_, followed by measuring the absorbance of the sample at 510 nm using catechin as the standard. This method, in addition to most flavonoids, also detects other phenolics containing a catechol group but, to simplify, in the text we refer to the AlCl_3_-reactive compounds collectively as ‘antioxidant flavonoids’ or simply ‘flavonoids’, and express their concentration in catechin equivalents (mg eq. C g^-1^ DW).

### Antioxidant enzyme activities

Crude protein extracts were prepared from frozen leaf tissue as described [28]. Protein concentration in the extracts was determined by the method of Bradford [29], using the Bio-Rad commercial reagent and bovine serum albumin (BSA) as a standard. The specific activity of superoxide dismutase (SOD), catalase (CAT), ascorbate peroxidase (APX), and glutathione reductase (GR), was measured in the protein extracts.

SOD activity was measured by following spectrophotometrically (at 560 nm) the inhibition of nitroblue tetrazolium (NBT) photoreduction, in reaction mixtures containing riboflavin as the source of superoxide radicals [30]. One SOD unit was defined as the amount of enzyme causing 50% inhibition of NBT photoreduction. CAT activity was determined as previously described [31], following the decrease in absorbance at 240 nm which accompanies the consumption of H_2_O_2_ added to the extract. One CAT unit was defined as the amount of enzyme that will decompose 1 μmol of H_2_O_2_ per minute at 25°C. APX activity was calculated by measuring the decrease in absorbance at 290 nm as ascorbate becomes oxidized in the reaction [32]. One APX unit was defined as the amount of enzyme required to consume 1 μmol of ascorbate per minute, at 25°C. GR activity was determined following the oxidation of NADPH (the GR cofactor in the reduction of oxidized glutathione) by the decrease in absorbance at 340 nm [33]. One GR unit was defined as the amount of enzyme that will oxidize 1 μmol of NADPH per minute at 25°C. Minor modifications to the original enzymatic assays have already been described [28].

### Statistical analyses

The program Statgraphics Centurion XVI was used for the statistical analysis of the experimental data. Before the analysis of variance, the validity of normality assumption was checked by the Shapiro-Wilk test, and the homogeneity of variance using the Levene test. Once it was established that ANOVA requirements were fulfilled, the significance of the differences among salt treatments was tested by one-way ANOVA at a 95% confidence level, while the Tukey HSD test was used to make post-hoc comparisons; ANOVAs were performed independently for the 15-day and 30-day treatments (and also independently for roots and leaves in the case of ion contents). Student’s t-tests at a 95% confidence level were employed to assess the significance of differences in pair-wise comparisons between the two harvest times (15 and 30 days) for each salt and water deficit treatment. All mean values mentioned throughout the text include the standard error (SE). In addition, all measured parameters in plants treated with NaCl for 15 and 30 days, were correlated using principal component analysis (PCA), separately for all variables related to growth parameters, photosynthetic pigments, ions and osmolytes, on the one side, and those related to oxidative stress and antioxidant systems, on the other.

## Results

### Stress-induced inhibition of plant growth

**Fig 1:**
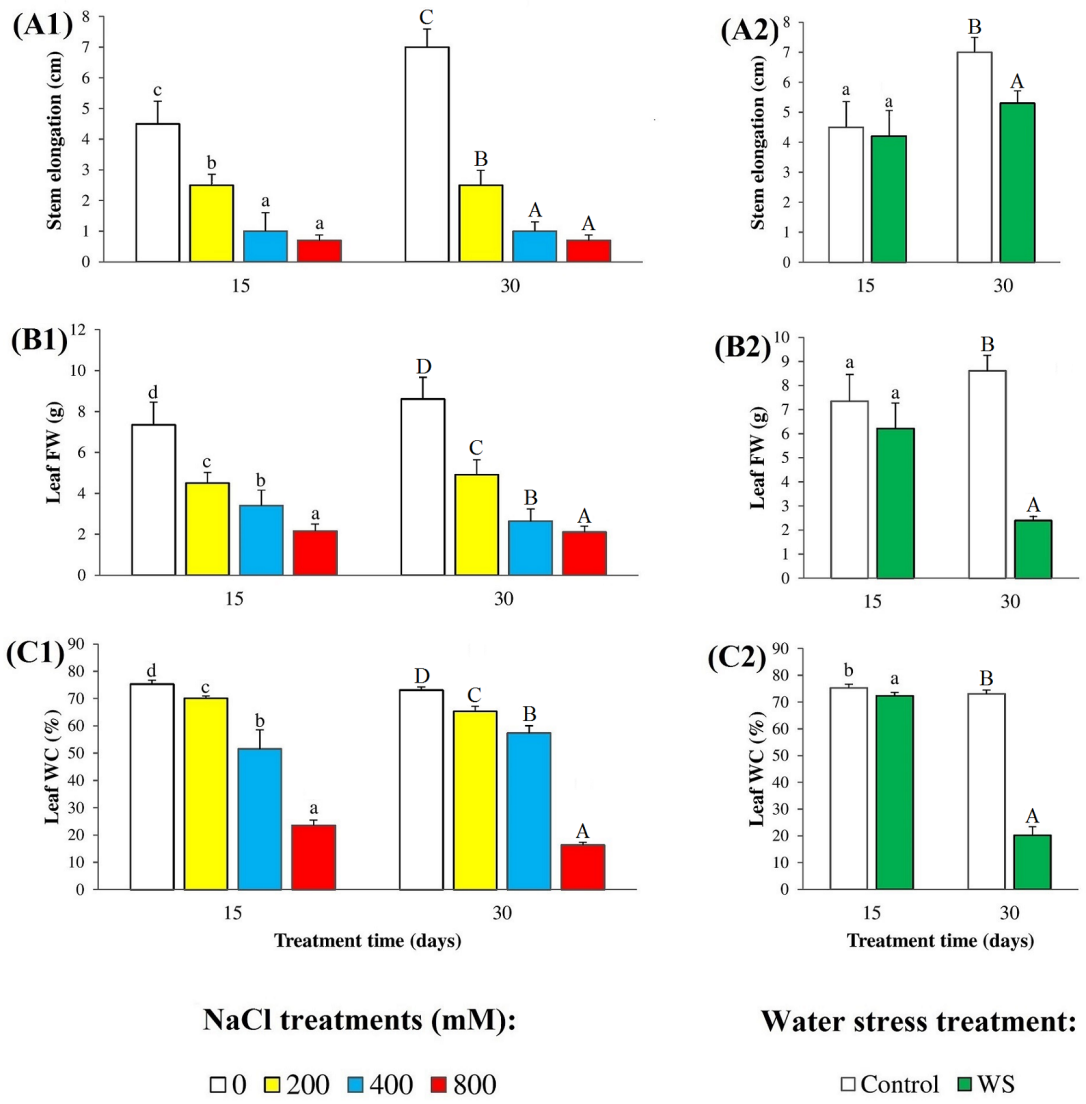
Stress-induced growth inhibition. Reduction of (**A**) stem length elongation (cm), (**B**) leaf fresh weight (FW) (g), and (**C**) leaf water content percentage (WC%), in oleander seedlings after 15 and 30 days of (**1**) salt treatments with the indicated NaCl concentrations, and (**2**) complete withholding of watering. Bars represent means with SE (n = 5). Different letters above the bars (lowercase for 15 days and capital for 30 days treatments) indicate significant differences between salt or water stress treatments, according to Tukey’s test (α = 0.05).

The exposure of *N*. *oleander* plants to salt resulted in a progressive, concentration-dependent inhibition of growth, in relation to the non-stressed controls, as shown by a relative reduction in stem elongation (Fig 1A1) and leaf FW (Fig 1B1) with increasing external NaCl concentrations. Changes in both growth parameters followed the same patterns in the 15 day and 30 day treatments, although salt effects appeared to be somewhat stronger in the longer treatment, at least considering stem length elongation: in all cases, values measured at 15 and 30 days were significantly different for all NaCl concentrations, while leaf FW reductions were similar, down to 25–30% of the corresponding controls at the highest NaCl concentration tested (800 mM) (Fig 1A1 and 1B1). Water stress, applied for 15 days did not have any significant effect on plant growth, either in terms of stem elongation or concerning leaf FW. Significant reductions of stem elongation—by ca. 30% of the control—and of biomass accumulation—by more than 70%—were only observed after 30 days without watering the plants (Fig 1A2 and 1B2). Oleander plants appeared to be relatively resistant to stress-induced leaf dehydration, showing very small (although statistically significant) reductions of WC in the presence of 200 mM NaCl (Fig 1C1) or after 15 days without water (Fig 1C2), by less than 10% in both cases. Substantial drops in WC were however observed in response to the strongest stress conditions tested—800 mM NaCl or 30 days of water stress—down to 31% and 22% of the corresponding controls in plants treated with salt for 15 and 30 days, respectively (Fig 1C1), and to 28% of the control in water-stressed plants after 30 days of treatment (Fig 1C2). In these treatments, therefore, the reduction of leaf FW is not only due to inhibition of growth but also to water loss.

### Degradation of photosynthetic pigments

**Fig 2:**
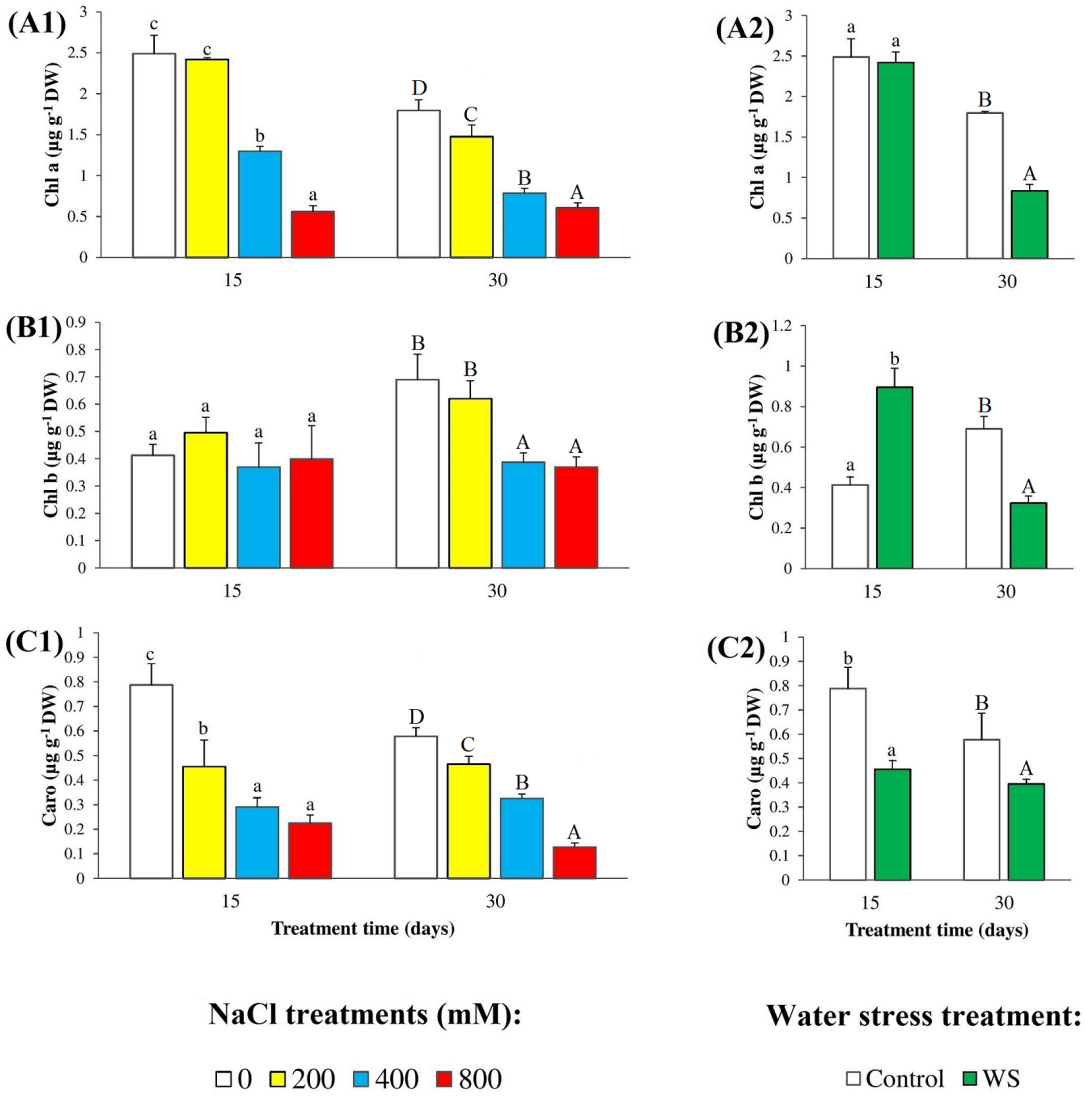
Degradation of photosynthetic pigments. Leaf concentrations of (**A**) chlorophyll a, (**B**) chlorophyll b and (**C**) total carotenoids, in oleander seedlings after 15 and 30 days of (**1**) salt treatments with the indicated NaCl concentrations, and (**2**) complete withholding of watering. Bars represent means with SE (n = 5). Different letters above the bars (lowercase for 15 days and capital for 30 days treatments) indicate significant differences between salt or water stress treatments, according to Tukey’s test (α = 0.05).

A reduction in the levels of photosynthetic pigments (chlorophyll a and b, and carotenoids) is a common effect of abiotic stress on plants, which we have also observed in our experiments with *N*. *oleander* (Fig 2). Control plants, grown for 15 days from the beginning of the stress treatments had higher leaf levels of chlorophyll a (Chl a) than those harvested after 30 days (ca. 2.5 and 1.8 μg g^-1^ DW, respectively), and in both cases Chl a contents decreased with the increase of external salinity, especially at high (400–800 mM NaCl) salt concentrations (Fig 2A1). Similarly, Chl a levels did not show any significant decrease in leaves of oleander plants subjected to 15 days of water stress but were reduced by more than 50% in 30 days-treated plants (Fig 2A2). These patterns were different for stress-induced changes of Chl b contents, which remained virtually unchanged after 15 days of salt treatments, at all tested NaCl concentrations; these values were significantly higher in control and 200 mM NaCl-treated plants in the second harvest, 30 days after starting the treatments, but decreased at 400 and 800 mM NaCl (Fig 2B1). Concerning Chl b levels in leaves of plants undergoing water stress, a twofold increase over the control was detected at 15 days of treatment, and a 50% decrease after 30 days (Fig 2B2). Both stresses caused significant reductions in Caro concentrations in the leaves, with similar qualitative patterns at 15 and 30 days of treatment (Fig 2C1 and 2C2).

### Monovalent ions levels

Concentrations of sodium (Na^+^) and chloride (Cl^-^) in roots and leaves of *N*. *oleander* increased in parallel with external salinity and in a time-dependent manner; that is, levels of both ions in roots or leaves were higher in the second harvest of plant material, for each NaCl concentration tested. The maximum relative increase in ion levels (comparing 800 mM NaCl-treated plants with the corresponding controls) was in all cases higher for Cl^-^ than for Na^+^, in leaves than in roots, and in the longer salt treatment (Table 1). When comparing Na^+^ and Cl^-^ contents in roots and leaves, a similar qualitative pattern of salt-induced variation was observed for both ions, albeit with quantitative differences between ions or treatments. At moderate salinity levels (200 mM NaCl), concentrations of Na^+^ and Cl^-^ were substantially higher—between 3-fold and 4-fold—in roots than in leaves. At 400 mM NaCl these differences were smaller (below 1.5-fold) or even non-significant. However, in the presence of 800 mM external NaCl ion concentrations measured in leaves were significantly higher, up to twofold of those determined in the roots (Table 1). Water stress treatments did not change significantly the levels of Na^+^ or Cl^-^ in roots or leaves, as it should be expected, except for a small reduction of Na^+^ contents in leaves of *N*. *oleander* plants water-stressed for 30 days.

**Table 1:** Monovalent ion contents in roots and leaves of *N*. *oleander* after 15 and 30 days of salt and water-stress treatments. Values shown are means (n = 5) ± SE.

Ions	Treatment time (days)	NaCl treatment (mM)	*N*. *oleander*		Water-stress treatment	*N*. *oleander*	
			Roots	Leaves		Roots	Leaves
Na^+^(μmol g^-1^ DW)	15	0	182.23 ± 12.27^a^	150.87 ± 9.25^a^	Control	182.23 ± 12.27^a^	150.87 ± 9.25^a^
		200	447.68 ± 22.28^b^	153.30 ± 17.43^a^	WS	160.03 ± 15.01^a^	161.53 ± 22.96^a^
		400	645.53 ± 60.80^c^	440.06 ± 23.05^b^			
		800	866.25± 62.31^d^	1635.22 ± 216.00^c^			
	30	0	156.95 ± 12.19^A^	155.74 ± 10.10^A^	Control	156.95 ± 12.19^A^	155.74 ± 10.10^B^
		200	668.38 ± 32.25^B^	156.95 ± 13.37^A^	WS	135.04 ± 12.68^A^	138.31 ± 9.95^A^
		400	873.36 ± 31.93^B^	740.27 ± 146.00^B^			
		800	1398.99 ± 200.44^C^	1981.04 ± 335.00^C^			
Cl^-^ (μmol g^-1^ DW)	15	0	143.86 ± 16.21^a^	105.78 ± 14.45^a^	Control	110.08 ± 27.61^a^	105.78 ± 14.45^a^
		200	406.20 ± 37.20^b^	148.92 ± 36.55^a^	WS	101.52 ± 11.97^a^	115.09 ± 21.37^a^
		400	677.01 ± 88.21^b^	575.45 ± 55.23^b^			
		800	994.35 ± 103.96^c^	1891.40 ± 156.40^c^			
	30	0	110.01 ± 27.61^A^	84.62 ± 15.71^A^	Control	143.86 ± 16.20^A^	84.62 ± 15.17^A^
		200	688.29 ± 83.20^B^	172.63 ± 9.89^B^	WS	93.78 ± 9.30^A^	94.78 ± 15.73^A^
		400	925.24 ± 58.97^B^	1091.68 ± 245.70^C^			
		800	1997.18 ± 276.10^C^	3884.34 ± 212.46^D^			
K^+^(μmol g^-1^ DW)	15	0	396.92 ± 37.39^b^	394.62 ± 32.85^a^	Control	396.92 ± 37.39^a^	394.62 ± 32.85^a^
		200	306.87 ± 9.08^ab^	360.13 ± 15.86^a^	WS	258.05 ± 15.98^a^	416.92 ± 29.16^a^
		400	318.28 ± 22.46^ab^	335.27 ± 48.43^a^			
		800	249.16 ± 10.27^a^	370.78 ± 37.19^a^			
	30	0	355.90 ± 22.08^C^	425.06 ± 27.15^B^	Control	355.90 ± 22.08^A^	425.06 ± 27.15^A^
		200	283.19 ± 5.92^B^	341.87 ± 24.37^A^	WS	369.43 ± 39.59^A^	392.60 ± 29.16^A^
		400	265.44 ± 8.33^B^	398.68 ± 22.17^AB^			
		800	173.28 ± 5.92^A^	334.26 ± 19.25^A^			

Different superscript letters (lowercase for 15 days and capital for 30 days treatments) indicate statistically significant differences between means (calculated independently for each ion, for roots and leaves and for the two treatment times), as established by one-way ANOVAs (salt treatments) or Student’s t-tests (water stress treatments) at a 95% confidence level.

Potassium (K^+^) contents showed a tendency to decrease, in roots and leaves, by increasing the external salinity, but the differences were statistically significant only in the plants subjected to the longer salt stress treatment, 30 days. As for Na^+^ and Cl^-^, K^+^ contents did not change with respect to the controls in water-stressed plants (Table 1).

**Fig 3:**
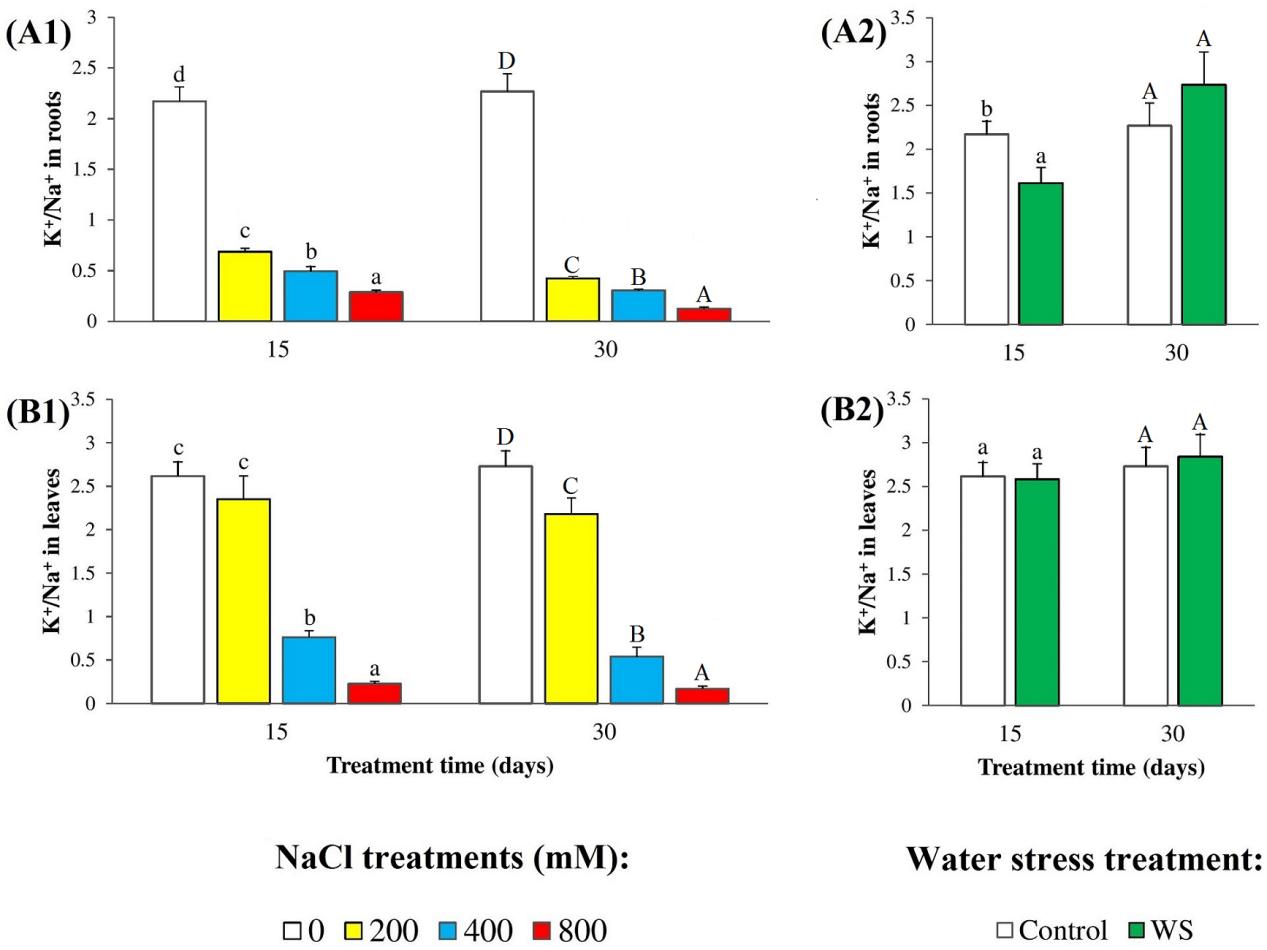
Potassium/sodium ratios. K^+^/Na^+^ ratios, in (**A**) roots and (**B**) leaves in oleander seedlings after 15 and 30 days of (**1**) salt treatments with the indicated NaCl concentrations, and (**2**) complete withholding of watering. Bars represent means with SE (n = 5). Different letters above the bars (lowercase for 15 days and capital for 30 days treatments) indicate significant differences between salt or water stress treatments, according to Tukey’s test (α = 0.05).

The relative changes of Na^+^ and K^+^ levels led to a clear salt-induced, concentration-dependent decrease of K^+^/Na^+^ ratios in relation to the non-stressed controls. This effect was stronger in the second harvest as compared to the first one, and in roots than in leaves. The reduction of K^+^/Na^+^ ratio in leaves in the presence of 200 mM NaCl was only 20% of the control after 30 days of treatment, and non-significant after 15 days; under the strongest salt stress conditions tested (30 days in the presence of 800 mM NaCl), K^+^/Na^+^ ratios decreased by more than 90% of the controls (Fig 3A1 and 3B1). K^+^/Na^+^ ratios decreased significantly in root samples of plants subjected to water deficit for 15 days (Fig 3A2), but no changes were detected after 30 days (Fig 3A2) or in leaves at both times (Fig 3B2).

### Osmolytes accumulation

**Fig 4:**
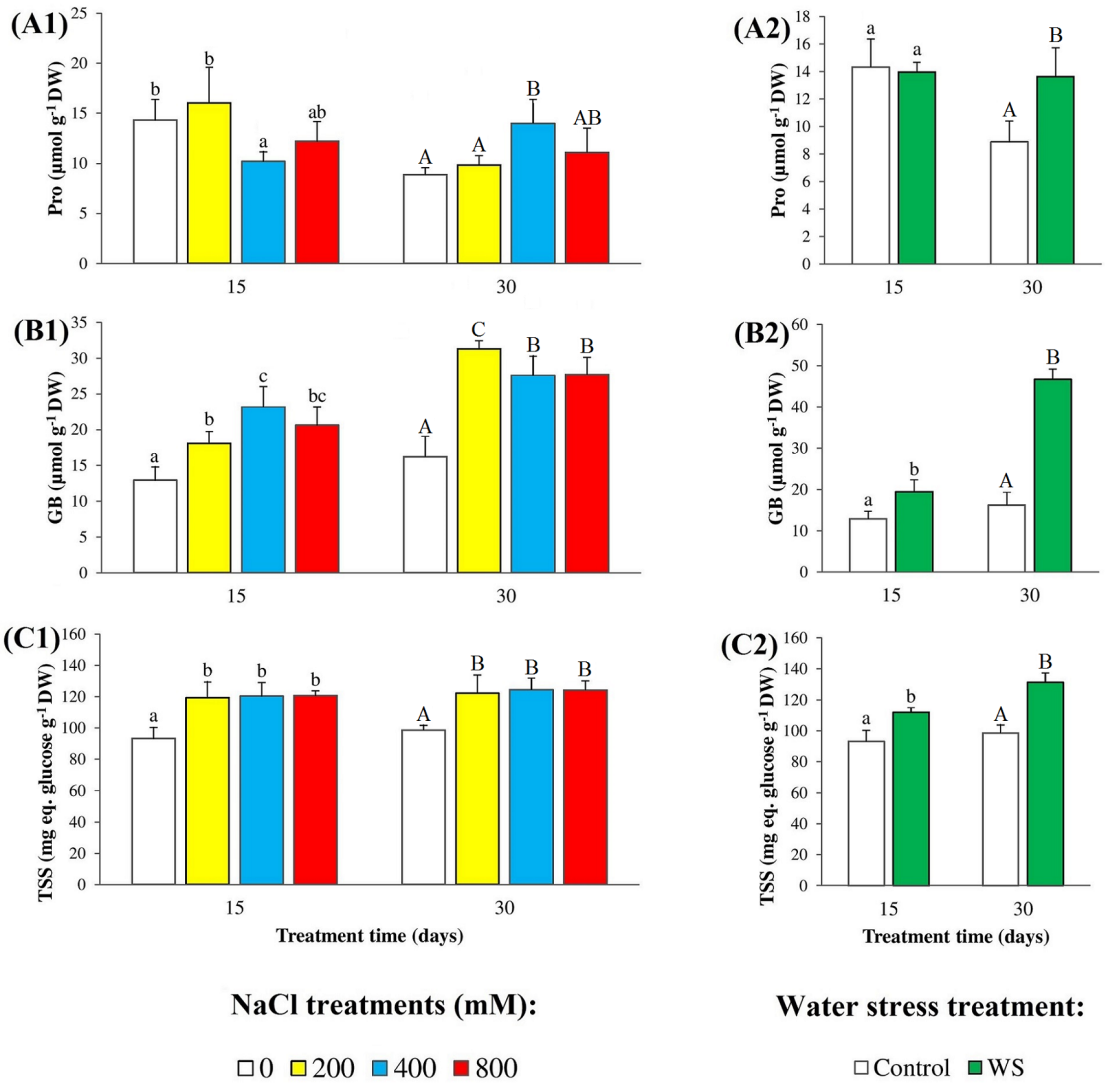
Osmolyte contents. Leaf contents of (**A**) proline (Pro), (**B**) glycine betaine (GB), and (**C**) total soluble sugars (TSS), in oleander seedlings after 15 and 30 days of (**1**) salt treatments with the indicated NaCl concentrations, and (**2**) complete withholding of watering. Bars represent means with SE (n = 5). Different letters above the bars (lowercase for 15 days and capital for 30 days treatments) indicate significant differences between salt or water stress treatments, according to Tukey’s test (α = 0.05).

High salinity and water deficit both cause osmotic stress in plants, which respond with the synthesis and accumulation of compatible solutes or osmolytes to help cellular osmotic adjustments. We have measured the levels of three types of osmolytes commonly used by plants: proline (Pro), glycine betaine (GB), and total soluble sugars (TSS), in the leaves of *N*. *oleander* plants subjected to salt and water stress treatments (Fig 4). Absolute Pro contents were found to be very low (less than 15 μmol g^-1^ DW in all cases) and, in general, did not change significantly, or increased little in response to increasing salinity or under water stress conditions (Fig 4A1 and 4A2). GB concentrations were also low in leaves of non-stressed plants, but increased up to almost twofold of the control in plants treated for 15 days with increasing salt concentrations; absolute GB levels were somewhat higher in the longer treatment (30 days), but the maximum relative increase was similar (Fig 4B1). Water stress also induced GB accumulation in leaves, with stronger time dependence as compared to salt: GB levels increased 1.5-fold over the control in plants harvested after 15 days of water deficit, and almost 3-fold in the second harvest at 30 days (Fig 4B2). The highest contribution to osmotic balance in oleander leaves seemed to be provided by TSS, which accumulated to much higher levels (in terms of glucose equivalents) than Pro and GB, even in the control plants. Increasing salinity and water deficit slightly increased TSS contents, between 1.2-fold and 1.3-fold in all assayed conditions (Fig 4C1 and 4C2).

### MDA and non-enzymatic antioxidants

**Fig 5:**
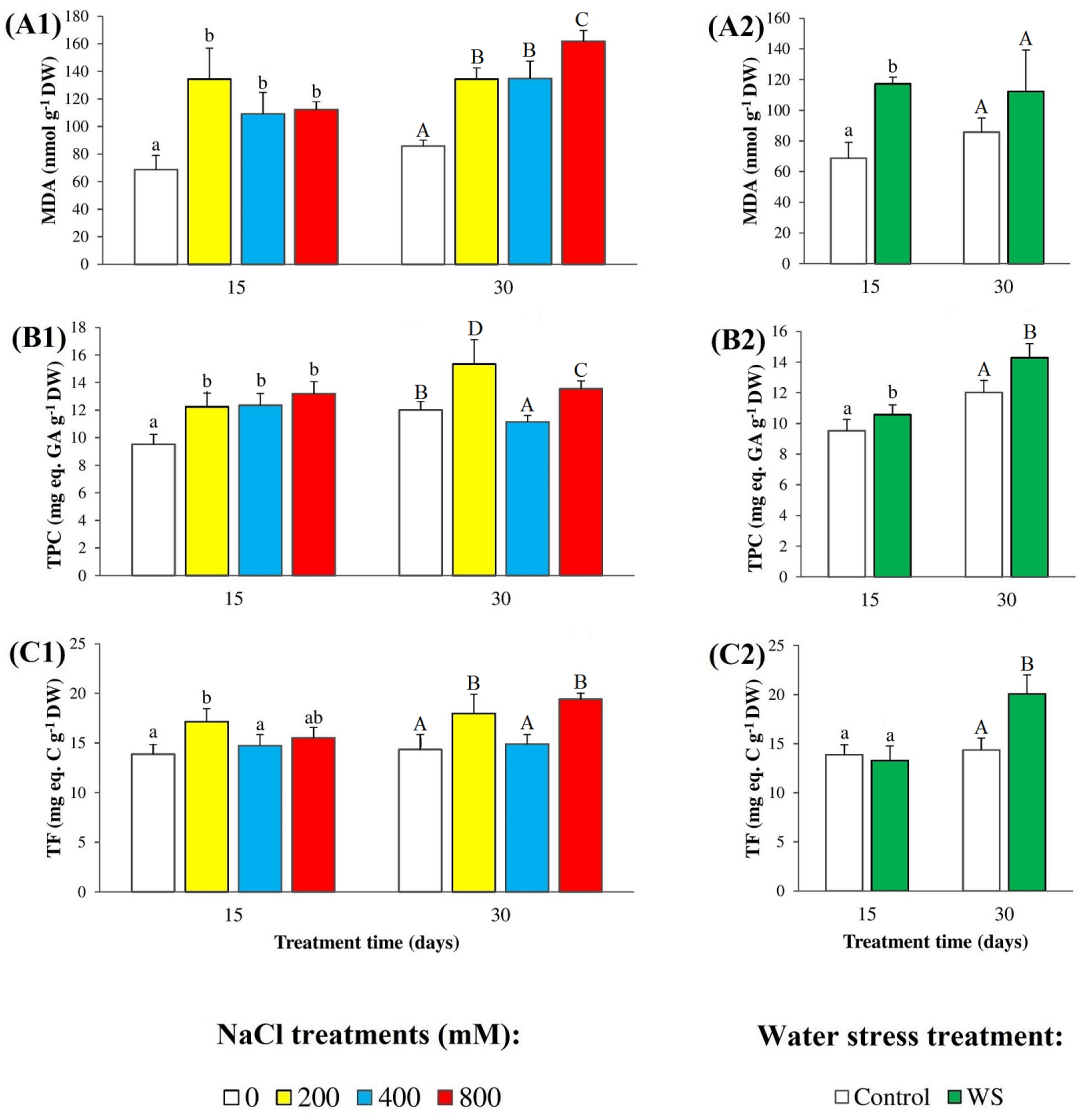
Oxidative stress and non-enzymatic antioxidants. Leaf levels of (**A**) malondialdehyde (MDA), (**B**) total phenolic compounds (TPC), and (**C**) total flavonoids (TF), in oleander seedlings after 15 and 30 days of (**1**) salt treatments with the indicated NaCl concentrations, and (**2**) complete withholding of watering. Bars represent means with SE (n = 5). Different letters above the bars (lowercase for 15 days and capital for 30 days treatments) indicate significant differences between salt or water stress treatments, according to Tukey’s test (α = 0.05).

The concentrations of malondialdehyde (MDA), a reliable biomarker of oxidative stress, and of total phenolic compounds (TPC) and total antioxidant flavonoids (TF), as examples of non-enzymatic antioxidants, were determined in the leaves of control and stressed *N*. *oleander* plants (Fig 5). A significant increase in MDA levels was detected in plants watered with saline solutions for 15 or 30 days, although the correlation with the applied salt concentrations was better in the latter case, reaching an almost twofold higher MDA content in the presence of 800 mM NaCl than in the control (Fig 5A1). Water deficit also caused oxidative stress in oleander seedlings, as shown by the increase in leaf MDA contents; this increase, however, was relatively lower than that observed in the presence of salt (Fig 5A2). A slight (yet statistically significant) increase in leaf TPC (Fig 5B1) and TF (Fig 5B2) concentrations was detected under salt stress, with increments ranging between 20% and 40% of the corresponding controls. Water stress induced a similar response, except that an increase of leaf TF levels was only detected after 30 days of treatment, but not in the first harvest at 15 days (Fig 5B2 and 5C2).

### Antioxidant enzymes activity

**Fig 6:**
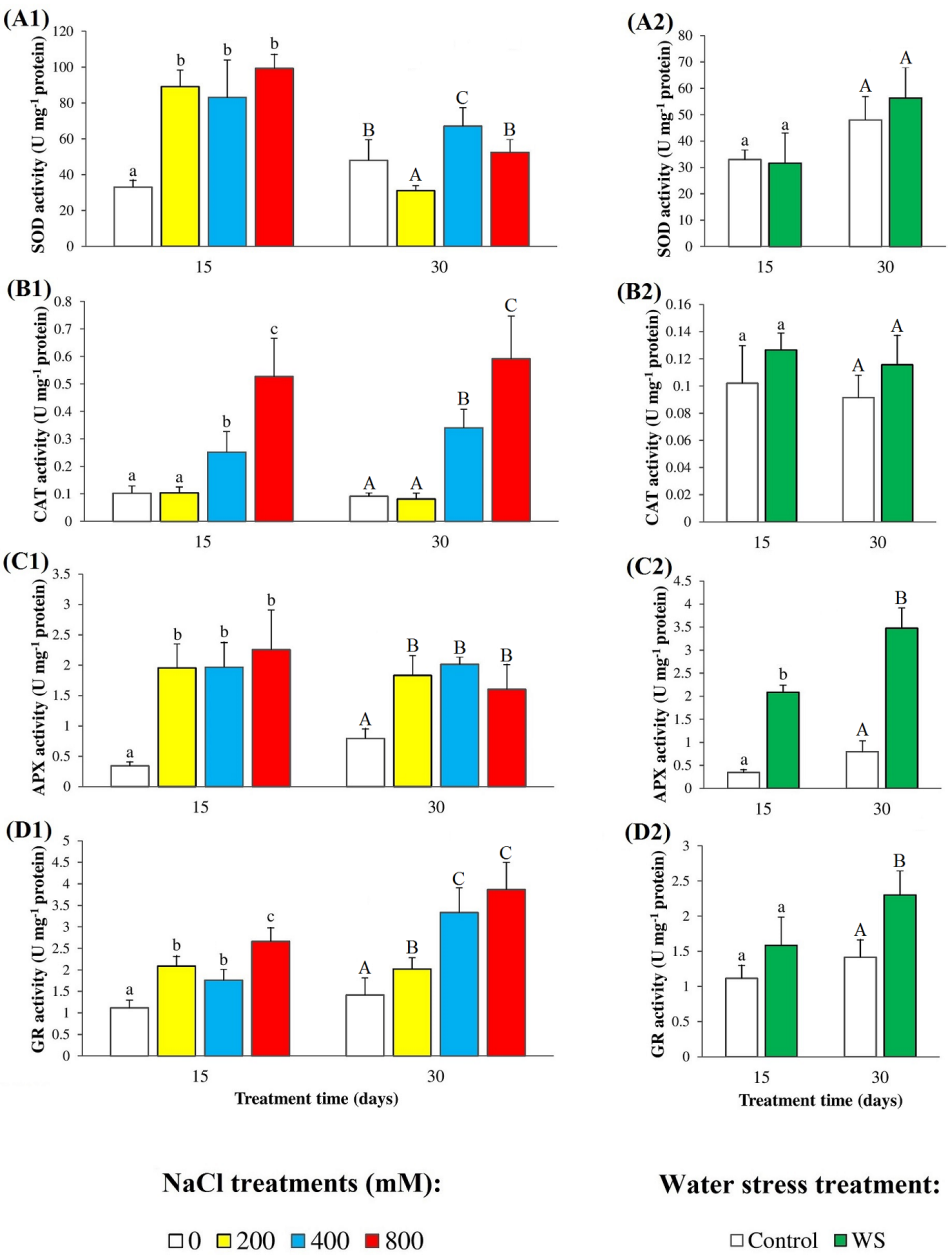
Antioxidant enzymes. Specific activities of (**A**) superoxide dismutase (SOD), (**B**) catalase (CAT), (**C**) ascorbate peroxidase (APX), and (**D**) glutathione reductase (GR), in leaves of oleander seedlings after 15 and 30 days of (**1**) salt treatments with the indicated NaCl concentrations, and (**2**) complete withholding of watering. Bars represent means with SE (n = 5). Different letters above the bars (lowercase for 15 days and capital for 30 days treatments) indicate significant differences between salt or water stress treatments, according to Tukey’s test (α = 0.05).

Activation of different antioxidant enzymes is also a general response of plants to the increase in the cellular levels of ‘reactive oxygen species’ (ROS) induced by drought, salinity and other abiotic stress conditions. We determined the specific activities of four of the most important antioxidant enzymes—superoxide dismutase (SOD), catalase (CAT), ascorbate peroxidase (APX), and glutathione reductase (GR)—in leaf extracts of control and stressed *N*. *oleander* plants (Fig 6). As a general picture, a significant increase of all four activities was observed in response to salt stress, but only in two of them (APX and GR) under water deficit conditions; yet, in several cases, the qualitative and quantitative patterns of stress-induced variations of enzyme activities were different for different enzymes and between the two treatment times. SOD specific activity increased about 3-fold over control values in leaves of plants grown for 15 days in the presence of salt, at all NaCl concentrations tested; after 30 days, SOD activity was higher in control plants, but the variations observed in salt-treated plants did not correlate with external salinity (Fig 6A1). Regarding CAT activity, it increased in the presence of 400 and 800 mM NaCl, in a concentration-dependent manner, with the same pattern and no significant differences between the samples harvested at 15 and at 30 days of treatment; the relative increase of CAT activity in relation to the controls was slightly higher for the longest treatment, 6.5-fold *vs*. 5.3-fold (Fig 6B1). APX specific activity also increased in salt-treated plants, without significant differences between the different treatments, regarding NaCl concentrations and treatment times; however, since APX activity of the control was lower at 15 days of treatment, the relative increase in leaf material collected in the first harvest was higher than in the second harvest (approximately 6.6-fold and 2.3-fold, respectively) (Fig 6C1). Finally, GR activities in leaf extracts showed concentration and time-dependent increases in response to increasing salinity, with the highest value (about 2.7-fold higher than in the corresponding control) measured in plants grown for 30 days in the presence of 800 mM NaCl (Fig 6D1). As mentioned above, SOD and CAT activities did not vary in water-stressed plants (Fig 6A2 and 6B2). Water deficit did induce an increase of APX activity, both in the first and second harvests (approximately 6.0-fold and 4.5-fold higher than in the corresponding controls, respectively) (Fig 6C2), while GR activity increased only slightly (1.6-fold) and only after 30 days of water deficit (Fig 6D2).

### Statistical analysis of the results

**Fig 7:**
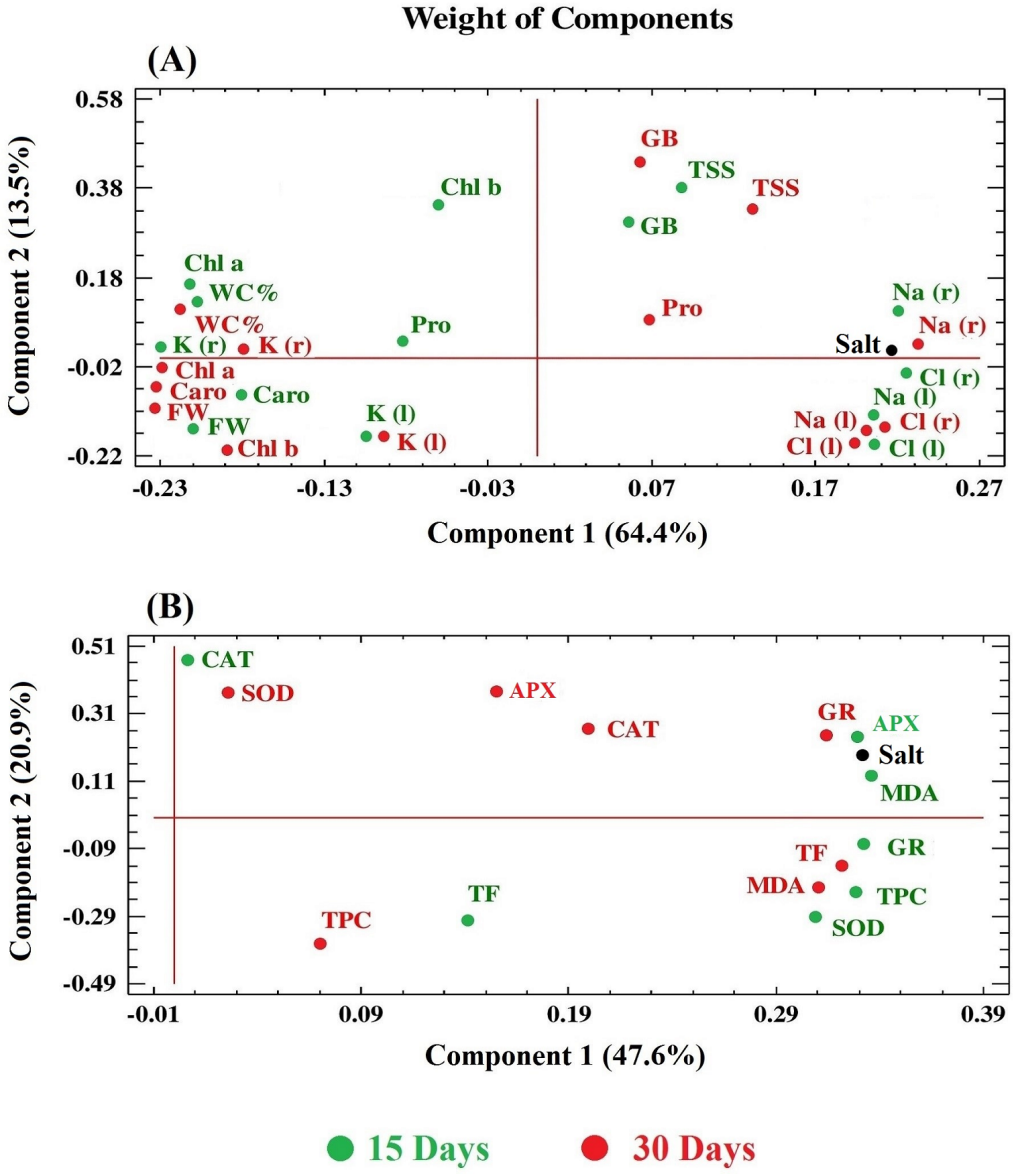
Principal Component Analyses (PCAs). Site score plot of the studied variables in the 15 days (green) and 30 days (red) salt stress treatments in *N*. *oleander*. PCAs included, as the analyzed variables: growth parameters, photosynthetic pigments, ions and osmolytes (**A**) or those related to oxidative stress and antioxidant systems (**B**). Symbols: FW, fresh weight; WC%, water content percentage; Chl a, chlorophyll a; Chl b, chlorophyll b; Caro, total carotenoids; Na^+^(r), sodium in roots; Na^+^(l), sodium in leaves; K^+^(r), potassium in roots; K^+^(l), potassium in leaves; Cl^-^(r), chloride in roots; Cl^-^(l), chloride in leaves; GB, glycine betaine; MDA, malondialdehyde; TPC, total phenolic compounds; TF, total flavonoids; SOD, superoxide dismutase; CAT, catalase; APX, ascorbate peroxidase; GR, glutathione reductase.

As a complement to the statistical analyses of the results of each set of experiments, correlations between all variables determined in plants submitted to salt stress treatments were established by performing two independent Principal Component Analyses (PCAs), the first one including those related to growth, photosynthetic pigments, ions and osmolytes (Fig 7A), and the second considering the oxidative stress marker (MDA) and antioxidant systems (Fig 7B). In the first PCA, two components had an Eigenvalue equal to or greater than 1 and explained a cumulative percentage of variance of 78%, approximately; the first component (X-axis), which alone explains 64% of the variation, is clearly determined by the salt treatment applied, as reflected by the very small angle of the ‘salt’ variable with this axis (Fig 7A). Variations of FW, WC% and photosynthetic pigments were found to be negatively correlated with the stress treatment, in agreement with the salt-induced inhibition of growth, leaf dehydration and degradation of chlorophylls and carotenoids; in all cases the correlation was stronger (variables closer to the X-axis) for the longer treatment (30 days). On the contrary, changes of Na^+^ and Cl^-^ concentrations, both in roots and leaves, correlated positively with the intensity of the applied stress; however, no clear differences were observed between the samples of the first and the second harvest, since the patterns of variation were maintained. Concerning K^+^ levels, a negative correlation with salinity, stronger in roots than in leaves, was detected in the samples harvested at 15 or 30 days of treatment, which can be explained by the observed reduction of K^+^ concentration in the presence of Na^+^. The vectors corresponding to osmolytes (Pro, GB, TSS) showed no or very weak correlation with salinity, reflecting the absence (Pro) or small increases (GB, TSS) in their concentration in response to salt stress; in fact, GB and TSS vectors appeared to be more related to the second component (Y-axis), which explains an additional 13% of the total variability (Fig 7A).

In the second PCA (Fig 7B), the first component, defined by the X-axis, indicated a strong positive correlation between the salt treatment and the degree of oxidative stress (variation of MDA levels), but explained only a 48% of the total variation [the second component (Y-axis) explained an additional 21%]. The results of this analysis showed a positive correlation of all variables (TPC and TF levels, antioxidant enzyme activities) with the salt treatment and oxidative stress, although with quantitative differences roughly corresponding to the results of the original experiments. Thus, TPC showed a stronger correlation at 15 days of treatment than in plants treated for 30 days, whereas the opposite was true for TF. For all enzymes except CAT, correlations with salinity were stronger in the first harvest than in the second—suggesting a partial inactivation of the enzymes when the stress treatment was prolonged, especially for APX and SOD. The vector corresponding to variation in CAT activity presented a smaller angle with the X-axis at 30 days than at 15 days, but in any case, the correlation was weaker than for the other enzymes (Fig 7B).

## Discussion

### Stress effects on plant growth and pigment contents

The first and most general response of plants to environmental stress conditions, such as drought or salinity, is inhibition of growth, as a consequence of the trade-off between the use of their resources (energy and metabolic precursors) for biomass accumulation and for the activation of defense mechanisms [16,34]. Salt and water deficit, as well as other stressful conditions, have a complex series of deleterious effects on plants contributing to growth inhibition, including for example disturbances in mineral nutrition, alteration of membrane permeability and of cellular osmotic balance, generation of oxidative stress by increasing reactive oxygen species (ROS) levels, or inhibition of different enzyme activities [15–17,35–37].

*Nerium oleander* follows the same qualitative patterns than other plant species in its responses to salt stress, which causes the inhibition of vegetative growth as shown by the relative reduction of fresh weight and stem elongation, in relation to control plants grown in the absence of salt. Oleander seems to be relatively more resistant to drought, as long (30 days) water stress treatments were necessary to detect a significant reduction of growth. Both, high salinity and water deficit in the soil impose an osmotic stress in plants, generally leading to leaf dehydration; yet oleander appears to be remarkably resistant to this osmotic effect, since substantial water loss (over 50%) was only detected in the presence of very high NaCl external concentration (800 mM) or after one month without any irrigation; that is, in response to extreme stress treatments; constitutive defense mechanisms, based on anatomic adaptations, are probably responsible for this behavior, at least partly [10,11].

A decrease in chlorophyll content due to salt stress has been observed in many herbaceous [38,39] or woody species [40,41], and appears to be a combined effect of the inhibition of enzymes such as Rubisco and PEP carboxylase, associated with chlorophyll biosynthesis [42], and the activation of chlorophyllase, involved in chlorophyll degradation [43]. A significant reduction in chlorophyll a contents was detected in oleander plants subjected to salt stress during 15 or 30 days, more clearly in the presence of high external salinities (≥ 400 mM NaCl); chlorophyll b also decreased in response to salt, but only after the longer treatment. Regarding the plants submitted to the drought treatment, only prolonged water stress reduced chlorophyll a and b levels. These data confirm the relative resistance of oleander to salt and, especially, drought stress. Carotenoids serve as accessory light harvesting compounds, which act transferring solar energy to chlorophylls, extending in this way the range of light wavelengths that can be used for photosynthesis. Carotenoids have been assigned additional roles in the defense mechanisms against stress, based on their antioxidant properties, for example as scavengers of singlet oxygen or protecting chlorophylls from the deleterious effects of photooxidation reactions [44,45]. Carotenoid levels were affected in oleander by salinity and by drought (30 days) as in many other species [46]. All comments above regarding the effects of increasing external salinity inhibiting plant growth and reducing photosynthetic pigment levels are fully supported by the PCA results, which showed a clear negative correlation of these parameters (FW, WC%, Chl a, Chl b, Caro) with the ‘salt treatment’ variable (X-axis), correlation that was stronger (the corresponding vectors had smaller angles with the X-axis) for the second harvest of plant material, after 30 days of treatment.

### Ion transport and ion homeostasis under stress conditions

Glycophytes, as well as monocotyledonous halophytes, cope with high salinity mostly by limiting Na^+^ and Cl^-^ uptake and transport to the plant leaves [47,48]. Dicotyledonous halophytes, on the other hand, possess efficient mechanisms to transport Na^+^ and Cl^-^ to the aerial part of the plant; in the leaves, these ions are predominantly accumulated in the vacuoles so that they do not reach toxic concentrations in the cytosol [4]. Therefore, concerning ion transport, oleander behaves as a typical glycophytic species, although the mechanisms blocking transport of toxic ions to the leaves appear to be much more efficient than in most non-halophytic species: Na^+^ and Cl^-^ concentrations are higher in roots than in leaves at moderate and even at relatively high salt concentrations (200–400 mM NaCl); only in the presence of extremely high external salinity—which the plant will never encounter in their natural habitats—those mechanisms break down and ions accumulate predominantly in the leaves. Ion transporters of the *HKT* gene family, which have been shown to play an important role in Na^+^ exclusion mechanisms [49], could be responsible for maintaining low leaf Na^+^ levels, but further studies will be required to establish the molecular basis of control of ion transport in oleander.

Increasing Na^+^ contents in plants normally leads to a reduction of K^+^ levels, since the two cations compete for the same binding sites and Na^+^ interferes with K^+^ uptake by using its physiological transport systems [47,50]; Na^+^ also induces the depolarization of the plasma membrane, activating outward-rectifying K^+^ channels and consequently causing an additional loss of cellular K^+^ [50–52]. Accordingly, a reduction of K^+^/Na^+^ ratios is generally observed under high salinity conditions, and mechanisms that help to maintain relatively high cellular K^+^/Na^+^ seem to be important for salt tolerance [53]. In our experiments with oleander, we detected relatively small reductions of K^+^ levels and K^+^/Na^+^ ratios in leaves of plants treated with 200 mM NaCl, in relation to the non-stressed controls, which could also contribute to the resistance of *N*. *oleander* to moderate salinity levels. In agreement with these data, the joint statistical analysis by PCA of the ‘ion contents’ variables showed highly significant correlations with the salt treatment, in roots and leaves, positive for Na^+^ and Cl^-^ (which increased with increasing salinity), and negative for K^+^ (which decreased as Na^+^ accumulated).

Ion transport does not seem to be involved in drought tolerance in oleander, since no significant variations were observed in Na^+^, Cl^-^ and K^+^ contents in *N*. *oleander* plants upon water deficit treatments.

### Osmolyte contents

Another common reaction to abiotic stress is the synthesis and accumulation of specific osmolytes in the stressed plants, although it is often difficult to establish whether the stress-dependent increase in the concentration of a particular osmolyte has a functional role in the mechanisms of tolerance of a given species. When accumulated at high enough levels, osmolytes will contribute to cellular osmotic adjustments under stress conditions and therefore to tolerance [54,55]. Yet osmolytes may have significant effects on tolerance even at much lower concentrations, due to their additional roles as low-molecular-weight chaperons and ROS scavengers, as well as participating in the regulation of gene expression and metabolic processes as signaling molecules [17,56,57].

Proline or glycine betaine are major osmolytes in many plant species, and their stress-induced accumulation represents an important mechanism of response to high salinity, water deficit and other abiotic stresses [17,54,55,58]. In oleander, however, Pro does not seem to be involved in stress tolerance, as no correlation of Pro contents with the level of stress was detected, apart from the fact that absolute Pro concentrations were very low, thus excluding any relevant osmotic effect. GB levels in control plants were also low, similar to those of Pro, but they increased significantly, up to more than twofold, in response to salt and water deficit; therefore, although osmotic effects are probably small, a contribution of this osmolyte to oleander stress tolerance cannot be ruled out.

Soluble sugars are also involved in the mechanisms of protection against abiotic stress in different plant species, and numerous publications report increases of total soluble sugars as a result of plant exposure to salt or water deficit [59,60]. The role of TSS as compatible solutes may be masked by their different additional functions in plants, as direct products of photosynthesis, components of primary metabolism and signaling and regulatory molecules, making sometimes difficult to assess their specific contribution to stress tolerance [61,62]. In oleander, TSS appear to be the major osmolytes, responsible for cellular osmotic balance; their levels increased only slightly in response to drought and salinity, but they were already high in control plants, suggesting the presence of constitutive mechanisms of response to stress in this species. Sucrose, glucose and fructose, together with some polyalcohols, are the commonest soluble carbohydrates accumulating in plant leaves under stress conditions [62], but fractionation of the leaf extracts and identification of individual sugars will be required to establish which specific sugars are the most relevant in oleander. The small (GB, TSS) or no increase (Pro) of osmolyte levels in response to salt stress explain the weak correlation of these variables with salinity observed in the PCA.

### Oxidative stress and activation of antioxidant systems

As reported for many other plant species [63–67], salt stress and water deficit induced oxidative stress in oleander, as shown by the increase of the leaf concentration of malondialdehyde (MDA), and the strong positive correlation between MDA and salinity observed in the PCA. MDA is a product of membrane lipid peroxidation and is considered as a reliable biochemical oxidative stress marker [68,69]. Different adverse environmental conditions increase the levels of ‘reactive oxygen species’ (ROS), therefore causing oxidative stress in plants, and plants respond by activating a battery of antioxidant systems, including several enzymes and different compounds with antioxidant activity [36,70,71]. Phenolic compounds, in general, and flavonoids, in particular, are secondary metabolites which act as strong antioxidants, and their accumulation in plants can reduce the oxidative damage caused, directly or indirectly, by abiotic stresses [72–74]. Flavonoids appear to represent a secondary ROS scavenging system, acting only when plants are affected by severe stress conditions and once primary antioxidant defence systems weaken [75]. Considering these facts, it was not surprising to detect significant increases of TPC and TF in leaves of stressed oleander plants, although with some qualitative and quantitative differences were observed between treatments.

The specific activities of the four antioxidant enzymes tested (SOD, CAT, APX and GR) increased significantly with increasing salinity, but only two of them (APX and GR) in response to water stress. It seems logical to conclude that activation of these enzymes will contribute to salt and drought tolerance in oleander, by counteracting one of the deleterious effects of stress, the increase in ROS levels. SOD is the primary defense against ROS, and acts eliminating superoxide radicals—specifically, dismutating two O_2_^•-^ radicals into H_2_O_2_ and O_2_ –which are precursors to other ROS and are generated in different subcellular compartments [76]. The increase in SOD specific activity has been attributed to the transcriptional activation of the SOS genes in the presence of superoxide; that is, to *de novo* synthesis of the enzyme [77]. As mentioned above, SOD specific activity increased in salt-treated plants, in a concentration-dependent manner, after 15 days of treatment; however, a relative reduction of this activity was observed after 30 days of exposure to high salt; this might be due to inactivation of the enzyme by H_2_O_2_ [78] or to disruption of the binding of the metal cofactor to the active centre of the enzyme [79]. CAT activity is induced by accumulation of its substrate, H_2_O_2_, which is decomposed by the enzyme to water and oxygen in peroxisomes and mitochondria [80]; CAT acts therefore after SOD, helping to reduce the levels of the generated H_2_O_2_. Probably this is the reason why CAT activation was observed only in the presence of relatively high salt concentrations (400–800 mM NaCl) and, consequently, why the PCA showed a weaker correlation of this activity with salinity and MDA levels. APX and GR also contribute to recover and maintain the appropriate cellular redox state in stressed plants, by catalyzing the reduction of H_2_O_2_ to water and oxygen coupled to ascorbate oxidation, and the reduction of oxidized glutathione (GSSG) to GSH using NADPH as a cofactor, respectively [81]. In our experiments, both enzymes were activated in response to salt stress; yet the relative increase in APX specific activity was higher after 15 days of treatment than in the second harvest at day 30, while the opposite was true for GR. This suggested that APX (as SOD) was partly inactivated upon prolonged salt treatment; indeed, in the PCA the two activities showed stronger correlations with salinity and MDA levels in the shorter treatment.

In the case of oleander’s responses to water deficit, considering the tested enzymes, activation of APX and GR appear to be enough to counteract or limit the negative effects of drought-induced oxidative stress. Since the species is relatively more resistant to water deficit than to salinity, probably it does not require triggering the activation of additional antioxidant enzymes, such as SOD or CAT, as a mechanism of defense against drought.

## Conclusions

The work described here confirms the high resistance of *Nerium oleander* to drought, which appears to be mostly based on constitutive defense mechanisms; they include anatomic adaptations of the leaf structure, which help to reduce leaf dehydration under water deficit conditions, and the accumulation of relatively high concentrations of soluble sugars for osmotic balance, even in the absence of stress. Moreover, activation of APX and, especially, GR enzymatic activities, and accumulation of non-enzymatic antioxidants (TPC, TF) contribute to counteract the deleterious effects of drought-induced oxidative stress. Oleander, although cannot be considered as a halophyte, is nevertheless quite resistant to salt stress. The same constitutive and inducible mechanisms responsible for drought tolerance (plus the activation of SOD and CAT activities) should also contribute to salt tolerance in oleander. In addition, oleander possesses efficient mechanisms to block transport of toxic ions to the aerial part of the plant, which break down only at very high external salinity and probably represents the response to salt stress most relevant for tolerance.
